# Focusing on the Role of Natural Products in Overcoming Cancer Drug Resistance: An Autophagy-Based Perspective

**DOI:** 10.3390/biom12111565

**Published:** 2022-10-26

**Authors:** Jiaqi Yao, Chi Ma, Kaixuan Feng, Guang Tan, Qingping Wen

**Affiliations:** 1 Department of Anesthesiology, The First Affiliated Hospital of Dalian Medical University, Dalian 116011, China; 2 Department of Hepatobiliary Surgery, The First Affiliated Hospital of Dalian Medical University, Dalian 116011, China; 3 Department of Anesthesiology, The Affiliated Xinhua Hospital of Dalian University, Dalian 116021, China

**Keywords:** autophagy, natural products, tumors, resistance, therapy

## Abstract

Autophagy is a critical cellular adaptive response in tumor formation. Nutritional deficiency and hypoxia exacerbate autophagic flux in established malignancies, promoting tumor cell proliferation, migration, metastasis, and resistance to therapeutic interventions. Pro-survival autophagy inhibition may be a promising treatment option for advanced cancer. Furthermore, excessive or persistent autophagy is cytotoxic, resulting in tumor cell death. Targeted autophagy activation has also shown significant promise in the fight against tumor drug resistance. Several research groups have examined the ability of natural products (NPs) such as alkaloids, terpenoids, polyphenols, and anthraquinones to serve as autophagy inhibitors or activators. The data support the capacity of NPs that promote lethal autophagy or inhibit pro-survival autophagy from being employed against tumor drug resistance. This paper discusses the potential applications of NPs that regulate autophagy in the fight against tumor drug resistance, some limitations of the current studies, and future research needs and priorities.

## 1. Introduction

One of the most lethal threats to human life and health, malignant tumors, are increasing in mortality and morbidity. In 2020, there were approximately 19.3 million new cancer diagnoses and 9.9 million deaths worldwide [1]. Chemotherapy, radiation, immunotherapy, and targeted therapy are the primary therapy modalities for tumors and have demonstrated success. However, the development of drug resistance significantly impacts the therapeutic outcomes. Intricate pathways of resistance to cancer therapy exist. Membrane transport proteins, the tumor microenvironment, tumor stem cells, programmed cell death (PCD), DNA damage, and epigenetics are factors known to play key roles in developing resistance to cancer therapy [2]. In recent years, there has been a surge of interest in using autophagy to combat tumor drug resistance.

Autophagy, a critical mechanism for removing defective proteins and organelles from cells, is also considered a type of PCD, along with apoptosis and necrosis. Autophagy can be triggered by various stresses including nutritional deprivation, hypoxia, oxidative damage, and DNA damage [3]. In the early stages of cancer progression, autophagy suppresses tumor development, whereas in the later stages of cancer progression, autophagy promotes tumor growth, shields cancer cells during therapy, and induces drug resistance [4]. Autophagy has the ability to reverse tumor drug resistance [5,6,7]. Some preclinical and clinical investigations indicate that chloroquine (CQ) and hydroxychloroquine (HCQ), two autophagy inhibitors, can increase sensitivity to chemotherapy and synthetic medicines [8]. They prevent increased autophagic flux caused by tumor therapy, enhancing therapeutic efficacy. However, using CQ and HCQ is constrained by factors such as side effects, dose limits, and non-specificity. There is an immediate need to identify specific and safe autophagy modulators for tumor drug resistance.

Natural products (NPs) have demonstrated advantages of high efficacy and low toxicity in treating various diseases. They are progressively attracting significant attention in the therapy of cancer. In particular, “French”, “Italian”, and “Japanese” paradoxes have all been positively linked to these NPs. The so-called paradoxes refer to the fact that people in these nations consume a lot of fat, although exhibit relatively low rates of cardiovascular disease and cancer [9]. The traditional diet of the Mediterranean and Japanese regions, as part of a healthy lifestyle, is linked to a lower risk of chronic illnesses and cancer, according to a growing body of scientific research [10,11]. In particular, resveratrol, a NP with anti-cancer effects, is found in the wine drunk in considerable amounts by the French. For this reason, the French paradox might be partially explained by the consumption of wine [10]. Tomato sauce, high in the antioxidant lycopene, is a pizza essential in Italy. Lycopene, like resveratrol, may fight inflammation and cancer [10]. The prevalence of chronic illnesses and cancer in Japan may be lowered by consuming fish rich in omega-3 fatty acids [11]. Dietary and plant-derived NPs are thus being evaluated for possible roles in cancer-preventative and therapy strategies. Our review focused on several NPs that have not been covered thoroughly in earlier publications. Several previous reviews have focused on describing the modulatory effects of one or a class of NPs on autophagy or the potential to combat tumor drug resistance [12,13,14]. To the best of our knowledge, this is the first comprehensive review of the topic that incorporates alkaloids, terpenoids, polyphenols, and anthraquinones. Overall, we summarize the majority of NPs that can regulate autophagy to achieve precise autophagy regulation and fight against tumor drug resistance.

## 2. Methods

We searched the PubMed and Google Scholar databases for publications written in English and published between the years 2010 and the present. Included in the list of search terms were “autophagy”, “tumor”, and “drug resistance” as well as “alkaloids”, “terpenoids”, “polyphenols”, or “anthraquinones”. All original research was considered, including animal trials and/or in vitro experiments. Initially, detecting autophagic flux should include observing the cellular ultrastructure and identifying molecular markers. Regarding NP screening, we did not consider any NPs that did not overcome drug resistance by modifying autophagy. Moreover, autophagic cell death can only be identified if three requirements are met: (1) increased autophagic flux; (2) cell death that occurs independently of apoptosis; and (3) the ability to reverse cell death by inhibiting autophagy. Finally, the study’s experimental design and dependability are crucial criteria for evaluating the quality of the literature. The experimental design of the literature included at least two groups: combination treatment with NPs and anti-tumor drugs (research group) and a group of anti-tumor drugs alone (control group). In addition, this study builds on published research and did not involve any human or animal experiments. Therefore, ethical approval from an institutional review board was not necessary.

## 3. Mechanisms of Autophagy

Autophagy is a cellular self-protection system that preserves cell survival under varied stress circumstances by degrading and reusing cellular proteins and peptides. Autophagy may be classified into three types based on the biodegradation process: macroautophagy, microautophagy, and chaperone-mediated autophagy [15]. In recent years, accumulating studies have highlighted autophagy as a critical research mechanism in tumor biology, whose activation or inhibition plays a “double-edged sword” function in tumor progression and drug resistance, as discussed in a review by Chang et al. in 2020 [16]. They believe that manipulating autophagy-mediated resistance could represent a vast study arena for tumor therapy.

Macroautophagy is the most common and sophisticated type of autophagy. Previous studies discussed in this article have focused on macroautophagy [17]. Five steps of autophagy have been proposed: induction, nucleation, elongation, fusion, and degradation [18]. Briefly, the UNC-51-like kinase 1 and 2 (ULK1/2), autophagy-related gene 13 (ATG13), and FIP200 proteins comprise a multiprotein complex that is vital in the early stages of autophagic vesicle formation. The phosphatidylinositol 3-kinase (PI3K-III) complex, comprising Beclin1, ATG14, VPS34, and p150, contributes to membrane formation and nucleation. Finally, the ULK1/2 complex forms a pre-initiation complex with the PI3K-III complex. It binds to the complex composed of ATG12, ATG5, and ATG16L to promote the conversion of microtubule-associated protein light chain 3 (LC3)-I to LC3-II. LC3-II marks the formation of intact autophagic vesicles. The selective uptake and load degradation of autophagosome loads is made possible by LC3-II’s interaction with the autophagy substrate, p62. Autophagosomes, on the other hand, include LC3-II in their outer membranes, which aids in the elongation and closing of the membrane. As a result, autophagy markers such as LC3-II and p62 are often used to monitor autophagic flux [19].

Autophagy is strictly regulated by ATGs and various signaling pathways [20]. For example, mammalian targets of rapamycin (mTOR) and AMP-activated protein kinase (AMPK) maintain low levels of cellular autophagy under normal conditions by suppressing the action of the ULK1/2 complex. The phosphoinositide 3-kinase (PI3K)/protein kinase B (AKT) signaling pathway is critical for autophagy because it can effectively activate mTOR and inhibit autophagy. AMPK functions as an energy receptor and frequently works as a negative regulator of mTOR, inducing autophagy. Compared with AMPK, p38MAPK suppresses LC3-I to LC3-II conversion via phosphorylating ATG5 protein and may act as a negative autophagy regulator. Furthermore, p38MAPK can suppress ERK activation, thus impairing cellular autophagy. The potential function of reactive oxygen species (ROS) as aerobic metabolites, which are toxic to cells, and upstream signaling molecules of autophagy, is highlighted in the report [21]. P53 is also a key molecule in the autophagic cascade. It plays a dual role in autophagy by controlling how different signaling pathways work. Specifically, nuclear p53 induces ATG expression based on its transcriptional activity to activate autophagy. In contrast, cytoplasmic p53 inhibits autophagy by suppressing AMPK, activating mTOR, and inhibiting ROS formation [22]. Post-translational modifications change how ATGs work. These changes are also crucial for controlling autophagy. It is possible that enzymes such as protein kinases and phosphatases as well as acetylases and ubiquitinates change autophagy-related proteins in response to stressors. The most well-known class IIb deacetylase, histone deacetylase 6 (HDAC6), has been demonstrated to impede autophagy by deacetylating TFEB and FOXO1, two key players in the process [23]. Additionally, non-coding RNAs including circRNAs, lncRNAs, and microRNAs play a role in autophagy initiation and inhibition [24,25]. Moreover, autophagy and apoptotic signaling are interdependent and interact with each other. In cancer cells, autophagy and apoptosis may oppose or encourage each other. Autophagy and apoptosis regulatory factors include proteins such as P53, ATGs, Beclin1, and Bcl-2 family proteins. For instance, autophagy as a pro-cell death process may induce apoptosis when it is excessive or persistent. How apoptosis and autophagy are regulated has significant implications for cancer research and therapy [26].

## 4. Autophagy and Tumor

### 4.1. The Dual Role of Autophagy in Tumors

At different stages of tumor growth, autophagy may exert bipolar functions. Autophagy may prevent tumorigenesis by controlling the cell cycle, boosting the immune response, decreasing DNA damage, and maintaining genomic stability. However, autophagy can also increase malignant cell growth. The processes may include providing energy to tumor cells, increasing the stemness profile of cancer stem cells (CSCs), regulating unfolded protein responses, promoting epithelial–mesenchymal transition, and facilitating cancer cell adaptation to hypoxia, oxidative stress, and DNA damage [27,28,29,30]. In other words, autophagy is associated with practically all cancer-related signaling pathways. Furthermore, certain ambiguous and complex mechanisms are highlighted. A review by Wang et al. highlighted the dominant role of autophagy in pathogenic microbial interactions with human cancer and suggested that targeting autophagy or microbiota could be a potential anti-cancer strategy [28]. The function of autophagy in the interaction between tumor cells and the tumor immune microenvironment is compelling. Autophagy has the potential to reshape the relationship between glioblastoma (GBM) cells and the immune microenvironment [31]. Dormancy is a unique state in which tumor cells exist. Dormant cells have the ability to reactivate and retain malignant biological behavior when activated, linked to tumor recurrence and drug resistance. The data confirm that autophagy is a significant factor of tumor cell dormancy [32]. In summary, autophagy’s role in cancer is dynamic and contentious, and is partially reliant on time and the environment.

### 4.2. Autophagy and Tumor Drug Resistance

The most exciting feature of autophagy regulation is its ability to regulate drug resistance in tumors [33]. Chemotherapy, targeted therapy, immunotherapy, and radiotherapy are currently the most frequently used types of tumor therapy. However, doctors and researchers continue to face difficulties with the resistance of the majority of patients to chemotherapeutic drugs, immune checkpoint inhibitors, and targeted medicines [34]. Resistance mechanisms include the aberrant expression of membrane transport proteins such as ATP-binding cassette transport proteins and P-glycoprotein, altered drug metabolic pathways, DNA repair, autophagy, hypoxic tumor microenvironments, enhanced stemness of tumor stem cells, mutations in epidermal growth factor receptor genes, and T cell depletion [35,36].

Tumor cells frequently initiate pro-survival autophagy in response to the cytotoxicity of chemotherapeutic drugs. Despite the absence of large-scale clinical trials, autophagy may be a critical target for drug resistance in human cancers. CQ and HCQ are the most commonly used autophagy inhibitors and have been demonstrated to be the primary inhibitors of drug resistance in multiple basic studies [37,38]. Aga et al. observed that combination therapy with cisplatin and CQ decreased nasopharyngeal carcinoma cell viability and increased cell apoptosis. Mechanistically, chemotherapy increased Beclin-1 expression, whereas CQ had the reverse effect [39]. In addition, Wang et al. elucidated that CQ could enhance the sensitivity of GEM to gallbladder cancer, depending on the modulation of autophagy [40]. Imatinib (IM) is a tyrosine kinase inhibitor (TKI) that has shown clinical efficacy and a favorable safety profile when used to treat recurrent or metastatic gastrointestinal stromal tumors. However, 20–30% of patients develop autophagy-mediated resistance and do not react well to IM therapy. CQ improves IM sensitivity by inhibiting autophagy through the mitogen-activated protein kinase (MAPK)/extracellular signal-regulated kinase (ERK) pathway [41]. Moreover, a vast number of anti-tumor drugs including docetaxel [42], doxorubicin (DOX) [43], mitogen-activated protein kinases 1/2 (MEK1/2) inhibitors [44], and pabisterostat [45] have been demonstrated to have anti-cancer activity in human cancer and links to CQ.

However, the clinical efficacy of targeted autophagy is mixed. A phase I/II trial conducted in 2014 demonstrated that the combination of HCQ, radiation therapy, and temozolomide (TMZ) had no effect on the survival rates in patients with GBM [46]. In contrast, a clinical trial was conducted to determine the anti-cancer effect of CQ in combination with taxane or taxane-like chemotherapy in patients with breast cancer. The ORR was 45.16% greater than planned. Patients experienced an increase in progression-free and overall survival [47]. Clinical trials in pancreatic cancer (PC) have demonstrated that CQ enhances the clinical response to gemcitabine (GEM) [48]. In addition to clinical efficacy, adverse reactions to CQ and HCQ are a key rationale for limiting their use. According to a clinical study, the daily administration of 500 mg CQ had no inhibitory effect on breast cancer cell proliferation. However, nearly 15% of patients terminated therapy due to CQ-related adverse effects [49]. Consequences of CQ and HCQ use such as gastrointestinal responses, skin hypersensitivity, and retinal toxicity are always important limitations. It is worth mentioning that both CQ and HCQ are not specific autophagy inhibitors. They may accumulate in acidic cell compartments and impair lysosomal activity, affecting the activation of autophagy [50].

In conclusion, although targeted autophagy has demonstrated significant promise in the fight against tumor drug resistance, various issues now preclude its clinical application. Conducting an urgent search for precise and safe autophagy modulators is critical.

## 5. Natural Products Overcome Autophagy-Mediated Tumor Drug Resistance

Autophagy is an adaptive response that tumor cells depend on for survival, particularly during periods of chemotherapeutic or targeted drug therapy. Protective autophagy, activated by numerous signaling pathways, supplies nutrition to cancer cells to maintain their growth and migration while also making them resistant to therapy [51,52]. As a result, autophagy suppression is often exploited as a target to increase cancer cell susceptibility to medicines. Autophagy inhibitors such as CQ, HCQ, and 3-methyladenine increase tumor cell susceptibility to treatment by reducing autophagosome formation, preventing autophagosome fusion with lysosomes, decreasing lysosomal degradation function, and suppressing ATG expression. Lethal autophagy, on the other hand, may serve as an alternate cell death mechanism for apoptosis-deficient cancer cells. Some autophagy inducers such as rapamycin have improved drug sensitivity by triggering autophagic cell death [53].

Recently, NPs derived from plants have shown unique advantages in the management of cancer, particularly in the battle against drug resistance [54,55]. Reviewing the literature revealed that a subset of NPs causes apoptosis in tumor cells by suppressing pro-survival autophagy. A subset of NPs promotes toxic autophagy, which causes autophagic cell death. More intriguingly, certain NPs may both trigger toxic autophagy and suppress pro-survival autophagy. We are thus interested in the mechanism of action of NPs against drug resistance in tumors through the modulation of autophagy.

### 5.1. Natural Products as An Inhibitor for Protective Autophagy

**Figure 1:**
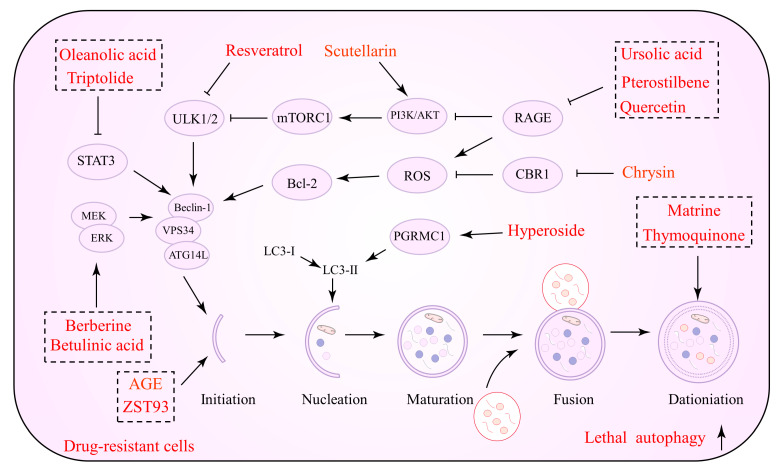
Natural products against tumor drug resistance by inhibiting pro-survival autophagy.

The inhibition of protective autophagy by NPs has significant advantages in inducing apoptosis in tumor cells, inhibiting their proliferation and reducing drug resistance. In advanced tumor stages, NPs inhibit the initiation and degradation processes of autophagy by suppressing autophagic vesicle formation or autolysosome formation, which in turn reverses drug resistance via the endogenous apoptotic pathway of cells. Several NPs that can be utilized to inhibit autophagy and promote cell apoptosis against drug resistance are listed in Table 1. Figure 1 depicts the precise process of the NP control of autophagy inhibition.

Plant-derived terpenoids are another possible novel anti-cancer drug class. They regulate cancer cell proliferation, migration, angiogenesis, and drug resistance [56]. Several terpenoids have been demonstrated to operate as autophagy modulators against tumor drug resistance and enhance chemotherapeutic and synthetic drug sensitivity [57]. Andrographolide (AG), a diterpene lactone from Andrographis paniculate, exerts anti-inflammatory, antiviral, and neuroprotective properties [58]. Data suggest that AG can be an essential anti-cancer agent, which should be introduced to oncology therapy to enhance chemotherapeutic sensitivity. Cisplatin, in conjunction with AG, increases the susceptibility of NSCLC cells to cisplatin, according to one study. AG reduces autophagic flux and limits the growth of drug-resistant cells by targeting the PTEN/AKT/mTOR pathway [59]. In lung cancer, AG promotes the conversion of LC3B-I to LC3B-II and reduces ATG5 protein expression, impeding autophagy. AG inhibits tumor cell growth and reduces the incidence of lung metastases when combined with cisplatin [60]. Notably, autophagy is not required for AG to improve VCR sensitivity. Essentially, AG regulates the PI3K/AKT/p53 signaling pathway, which promotes apoptosis rather than autophagy [61]. Consistent with this evidence, terpenoids such as α-hederin [62], jolkinolide B [63], PC3-15 [64], pristimerin [65], celastrol [66], and hemistepsin A (HsA) have been demonstrated to act against drug resistance by inhibiting autophagic fluxes and promoting cell apoptosis.

**Table 1:** Natural products that act against tumor drug resistance by inhibiting pro-survival autophagy.

Compounds	Plant Origin	Classification	Cancer Types	Models and Dosage	Mechanism	Main Effects	References
Andrographolide	Andrographis paniculata	Terpenoid	Non-small-cell lung cancer (NSCLC)	In vitro: A549/DDP cell line (30 µM) In vivo: mice (5 mg/kg)	Inhibits autophagy and promotes cell apoptosis	Facilitates cisplatin sensitivity	[59]
α-Hederin	Hedera helix	Terpenoid	NSCLC	NCI-H1299 and NCI-H1650 cell lines (12.5 µM, 24 h)	Inhibits autophagy and promotes ROS accumulation	Facilitates paclitaxel sensitivity	[62]
Jolkinolide B	Euphorbia fischeriana Steud	Terpenoid	Bladder cancer	In vitro: UM-UC-3 and T24 cell lines (2.5 or 5 µM, 48 h) In vivo: mice (intraperitoneally injected, 10 mg/kg/day)	Inhibits autophagy and promotes cell apoptosis	Facilitates temsirolimus, rapamycin, and everolimus sensitivity	[63]
PC3-15	Schisandra propinqua (Wall.) Baill. var. propinqua	Terpenoid	Breast cancer	In vitro: MDA-MB-468 and HEK293T cell lines (20 µM, 6 h) In vivo: mice (oral gavage, 50 mg/kg/day)		Facilitates lapatinib sensitivity	[64]
Pristimerin	Celastraceae/ Hippocrateaceae	Terpenoid	Lung cancer (LC)	In vitro: A549 and NCI-H446 cell lines (0.25 µM, 24 h) In vivo: mice (0.8 mg/kg/day)	Inhibits autophagy and promotes cell apoptosis	Facilitates cisplatin sensitivity	[65]
Celastrol	Tripterygium wilfordii Hook F	Terpenoid	LC	A549, HCC-15, and Calu-3 cell lines (1–4 µM, 12 h)	Inhibits autophagy and promotes cell apoptosis	Facilitates TRAIL sensitivity	[66]
Icariin	Epimedium brevicornum Maxim.	Polyphenol	Breast cancer	MCF-7 and T47D cell lines (10–75 µM, 24 h)	Inhibits autophagy and promotes cell apoptosis	Facilitates tamoxifen sensitivity	[67]
			Ovarian cancer	SKVCR cell line (10–75 µM, 24 h)	Inhibits autophagy and promotes cell apoptosis	Facilitates cisplatin sensitivity	[68]
Apigenin	Apiaceae	Polyphenol	Hepatocellular carcinoma (HCC)	In vitro: BEL-7402/ADM cell line In vivo: mice (intratumorally injected, 50 mg/kg/day)	Inhibits autophagy	Facilitates doxorubicin sensitivity	[69]
Tea polyphenol	Camellia sinensis	Polyphenol	Bladder cancer	T24 and BIU87 cell lines (100 µM, 24 h)	Inhibits autophagy and promotes cell apoptosis	Facilitates epirubicin sensitivity	[70]
Genistein	Soybeans and soy products	Polyphenol	LC	A549 cell line (40 µM, 12 h)	Inhibits autophagy and promotes cell apoptosis	Facilitates TRAIL sensitivity	[71]
Phloretin	Apples	Polyphenol	Breast cancer	In vitro: MCF7 and MDA-MB-231 cell lines (100–300 µM, 24 h) In vivo: mice (oral gavage, 100 mg/kg/day)	Inhibits autophagy	Facilitates TMX and DOX sensitivity	[72]
Formononetin	Astragalus membranaceus	Polyphenol	Breast cancer	In vitro: MDA-MB-231 cell line (15 µM) In vivo: mice (given orally, 30 mg/kg/3 day)	Inhibits autophagy and promotes cell apoptosis	Facilitates taxol sensitivity	[73]
Rutin	Potentilla discolor Bunge	Polyphenol	HCC	In vitro: HepG2 and HCCLM3 cell lines (75 µM, 24 h) In vivo: mice (intratumorally injected, 3 mg/kg/2 day)	Inhibits autophagy	Facilitates sorafenib sensitivity	[74]

Polyphenols are a class of secondary metabolites in plants that exhibit various pharmacological activities [75]. Notably, the impact of polyphenols in preventing drug resistance appears encouraging in oncology therapy [14]. Icariin is one of the main active ingredients of the Chinese botanical drug Epimedium, a well-researched anti-cancer agent [76]. Icariin induces apoptosis in TMX-resistant breast cancer cells and inhibits autophagy; thus, it is an ideal sensitizing agent. Combined treatment with autophagy inhibitors and icariin also induced potent antitumor effects, while tumor cells modified with overexpressing ATG5 were found to resist the toxic effects of icariin [67]. Indeed, ATG5 is also an essential player in the enhancement of cisplatin sensitivity by icariin. Icariin activates the AKT/mTOR/ATG5 signaling pathway in cisplatin-resistant OC cells, inhibiting autophagy and causing cell death [68]. Hypoxia has been implicated in the efficacy of anti-cancer medications as a critical factor that activates many drug resistance pathways in tumor cells [77]. Numerous correlations between drug resistance and hypoxia have been discovered. Hypoxia-inducible factor-1 (HIF-1) is an essential regulator of cellular adaptation to hypoxia; it is frequently overexpressed in cancer cells and has been related to drug resistance [78,79]. Apigenin (APG), a flavonoid, is a natural active ingredient that exhibits anti-cancer action as well as a favorable safety profile. APG can perturb the tumor cell microenvironment, induce ERS, and trigger autophagic cell death [80]. Among the APG targets, HIF-1α and Ezh2 have been identified. APG treatment may activate ERS signaling, increase the expression of autophagy-related proteins, and downregulate the expression of p-mTOR and p62. Notably, this effect was observed under normal and hypoxic conditions, indicating that APG does not exhibit selective behavior [81]. Drug resistance has also been hypothesized to be targeted via non-coding RNA-mediated autophagy. DOX-resistant HCC cells showed an ATG7-dependent autophagy pathway driven by miR-520b. In other words, protective autophagy promoted drug resistance in tumor cells, whereas miR-520 mimics inhibited ATG7-dependent autophagy against DOX resistance. In conjunction with DOX, APG stimulated miR-520b expression [69]. In conclusion, APG causes autophagic cell death in tumor cells, and by suppressing protective autophagy, it may increase chemotherapeutic drug susceptibility. Furthermore, several polyphenols such as tea polyphenol [70], genistein [71], apple dihydrochalcone phloretin [72], formononetin (FMNT) [73], and rutin [74], which reduce off-target effects and enhance drug sensitivity, are currently under investigation.

### 5.2. Natural Products as Promoters of Lethal Autophagy

**Figure 2:**
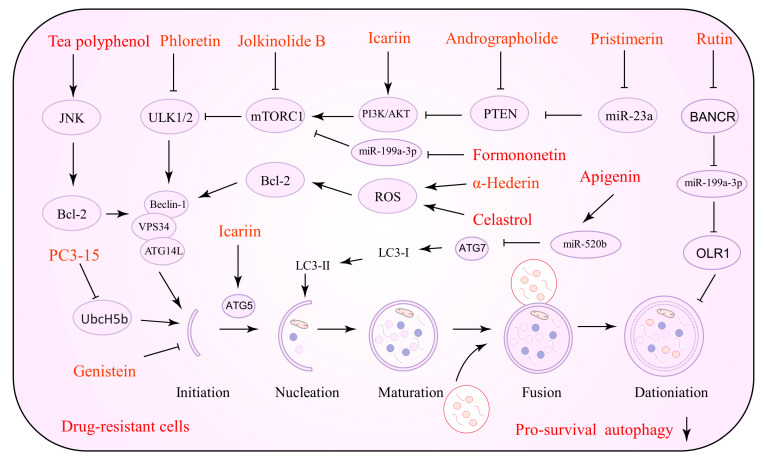
Natural products against tumor drug resistance by inducing lethal autophagy.

In vivo and in vitro studies have shown that NPs that promote lethal autophagy are useful against tumor treatment resistance. NPs activate cancer cell autophagy, improve chemotherapeutic drug sensitivity by increasing lysosomal membrane permeability, and induce tumor cell apoptosis by activating intact autophagic flow through several pathways including PI3K/AKT/mTOR, AMPK, and ROS [82,83]. Several NPs that can be utilized to induce lethal autophagy against drug resistance are listed in Table 2. Figure 2 depicts the precise process of the NP control of autophagy activation.

Alkaloids are NPs of importance with potential value in cancer therapy. Alkaloids have been demonstrated to impact malignant cancer cell proliferation and work in tandem with chemotherapeutic drugs. Berberine (BBR), as observed by Zhang and his group, downregulates the c-Myc signaling pathway and increases ROS production, which causes an increase in the sensitivity of lapatinib [84]. BBR is an alkaloid capable of upregulating basal autophagy and inducing hyperautophagy. Its properties make BBR essential for malignant proliferation and drug resistance in cancer research [85]. BBR may modulate autophagic flux and apoptosis in drug-resistant cells. In this sense, BBR downregulates the ERK1/2 signaling pathway that induces intracellular ROS accumulation and EGFR degradation, all of which have been linked to chemotherapy and targeted therapy sensitivity [86,87]. Matrine is an active component of the botanical drug Sophora flavescens, which exhibits potent anti-tumor properties. Matrine targets ATGs and affects the cell cycle of tumor cells, enhancing the sensitivity of drug-resistant cells to vincristine (VCR) and adriamycin (ADM) [88].

**Table 2:** Natural products that act against tumor drug resistance by inducing lethal autophagy.

Compounds	Plant Origin	Classification	Cancer Types	Models and Dosage	Mechanism	Main Effects	References
Berberine	Coptis chinensis	Alkaloids	Glioblastoma	In vitro: U87 and U251 cell lines (10 µM, 24 h) In vivo: mice (intraperitoneally injected, 50 mg/kg/day)	Induces autophagy and promotes cell apoptosis	Facilitates temozolomide sensitivity	[86]
			Non-small-cell lung cancer (NSCLC)	In vitro: H460 and H1299 cell lines (10, 25, 50 µM, 72 h) In vivo: mice (oral gavage, 80 mg/kg/day)	Induces autophagy and promotes cell apoptosis	Facilitates icotinib sensitivity	[87]
Matrine	Sophora flavescens	Alkaloids	Leukemia	K562/ADM cell line (0.5, 1, 2 mg/mL, 48 h)	Induces autophagy and promotes cell apoptosis	Facilitates vincristine and adriamycin sensitivity	[88]
Ursolic acid	Lamiaceae	Terpenoids	Pancreatic cancer (PC)	MIA Paca-2 cell line (50 μM, 24 h)	Induces autophagy and promotes cell apoptosis	Facilitates gemcitabine (GEM) sensitivity	[89]
Betulinic acid	Mirabilis jalapa	Terpenoids	Lung cancer (LC)	HCC827 and H1975 cell lines (20 µM, 48 h)	Induces autophagy and promotes cell apoptosis	Facilitates Iressa and Tarceva sensitivity	[90]
Triptolide	Tripterygium wilfordii	Terpenoids	PC	In vitro: MIA PaCa-2 and PANC-1 cell lines (50 nM, 48 h)	Induces autophagy and promotes cell apoptosis	Facilitates TNF-related apoptosis-inducing ligand (TRAIL) sensitivity	[91]
			Ovarian cancer	In vitro: SKOV3/DDP cell line (100 nM, 12 h) In vivo: mice (intraperitoneally injected, 0.15 mg/kg/day)	Induces autophagy and ROS accumulation	Facilitates cisplatin sensitivity	[92]
Oleanolic acid	Oleaceae	Terpenoids	Cervical cancer	In vitro: HeLa cell line (30 µM, 24 h) In vivo: mice (oral gavage, 10 and 40 mg/kg/day)	Induces autophagy	Facilitates cisplatin sensitivity	[93]
AGE	Sanguisorba officinalis L.	Terpenoids	Colorectal cancer	RKO-R, HCT15-R, RKO-P, and HCT15-P cell lines (10 and 20 µM, 24 and 48 h)	Induces autophagy and promotes cell apoptosis	Facilitates 5-FU sensitivity	[94]
Demethylzeylasteral	Tripterygium wilfordii Hook F	Terpenoids	PC	In vitro: MIA PaCa-2 and PANC-1 cell lines (0.1–1 pg/mL, 24–72 h) In vivo: mice (oral gavage, 80, 160, and 200 mg/kg/day)	Induces autophagy and promotes cell apoptosis	Facilitates GEM sensitivity	[95]
Resveratrol	Veratrum gandiflorum	Polyphenols	Oral cancer	CAR cell line (50 µM, 48 h)	Induces autophagy and promotes cell apoptosis	Facilitates cisplatin sensitivity	[96]
			Ovarian Cancer	SKOV3 and OVCAR3 cell lines (100 µM, 72 h)	Induces autophagy and promotes cell apoptosis	Facilitates platinum sensitivity	[97]
Pterostilbene	Resveratrol	Polyphenols	Pancreatic ductal adenocarcinoma	MIA PaCa-2 cell line (25, 50 µM, 72 h)	Induces autophagy and promotes cell apoptosis	Facilitates gemcitabine (GEM) sensitivity	[98]
Quercetin	Vegetables, fruits, and herbs	Polyphenols	PC	MIA Paca-2, BxPC-3, AsPC-1, HPAC, and PANC-1 cell lines (25–50 µM, 24 h)	Induces autophagy and promotes cell apoptosis	Facilitates GEM sensitivity	[99]
Hyperoside	Hypericum and Crataegus	Polyphenols	Ovarian cancer	SKOV-3 and HO-8910 cell lines (100 µM, 48 h)	Induces autophagy and promotes cell apoptosis	Facilitates cisplatin sensitivity	[100]
Scutellarin	Erigeron breviscapus Hand-Mazz	Polyphenols	NSCLC	In vitro: PC-9, H1975, and A549/DDP cell lines (120 µM, 24–48 h) In vivo: mice (oral gavage, 60 mg/kg/day)	Induces autophagy and promotes cell apoptosis	Facilitates cisplatin sensitivity	[101]
Chrysin	Passiflora caerulea	Polyphenols	PC	In vitro: ANC-1, Capan-2, BxPC-3 and AsPC-1 cell lines (50 µM, 24 h) In vivo: mice (oral gavage, 30 mg/kg/day)	Induces autophagy and promotes cell ferroptosis	Facilitates GEM sensitivity	[102]
Thymoquinone	Nigella sativa	Anthraquinones	Breast cancer	In vitro: MCF-7 and T47D cell lines (10–100 µM, 24–48 h)	Induces autophagy and promotes cell ferroptosis	Facilitates GEM sensitivity	[103]

Ursolic acid (UA) is a pentacyclic triterpenoid with a broad spectrum of anti-cancer properties. Lin et al. revealed that the UA treatment of GEM-resistant cells resulted in the activation of ERS and the suppression of receptors for advanced glycation end products (RAGE), concomitant to enhanced apoptosis and autophagy [89]. Toxic autophagy and ferroptosis were significantly elevated in osteosarcoma cells treated with cisplatin and UA [104]. As a result of these findings, UA may be an ideal adjuvant for chemotherapy drugs. In addition, one study found that betulinic acid (BA) could act against the acquired resistance of EGFR-TKI [90]. Compared with EGFR TKI alone, the combination of BA and EGFR TKI demonstrated promising efficacy against EGFR-TKI-resistant lung cancer cells and was associated with inducing cytotoxic autophagy. Additionally, BA blocked the ERK/MEK signaling pathway, promoted toxic autophagy, and enhanced autophagic cell death in SGC-7901 cells (GC lines) [105]. However, ERK inhibitors (ERKi) do not exploit the promotion of autophagy by BA for NSCLC therapy. Even if ERKi is present, NSCLC cells are more susceptible to BA. Dual treatment with BA and ERKi, on the other hand, resulted in an increase in protective autophagy, which significantly reduced the effectiveness. Notably, the addition of HCQ to dual treatment with BA and ERKi was more effective than either single or dual therapy [106]. In summary, this evidence suggests that combination therapy may provide clinical benefits for oncology patients as a potential treatment option, possibly reducing drug resistance.

Similarly, aloe emodin (AE), isolated from Rheum palmatum L., is a well-known anthraquinone compound that exerts significant anti-tumor effects [107]. Recent research has found that AE and targeted drug delivery systems significantly overcome drug resistance in various human cancers [108]. Interestingly, Cheng et al. found that AE also enhanced protective autophagy in reversing ADM-induced drug resistance. By supplementing AE with autophagy inhibitors, anti-tumor activity and sensibility may be enhanced (in vitro: MCF-7/ADR cell line, 20 μM, 48 h) [109].

Triptolide (TPL), a naturally occurring compound that acts via a distinct molecular mechanism, has shown growth inhibitory efficacy in preclinical studies for various solid tumors [110]. TPL may be an alternative for treatment-resistant human malignancies due to its high sensitivity and minimal toxicity [111]. The tumor-necrosis-factor-related apoptosis-inducing ligand (TRAIL) can form a homotrimer with its receptor to initiate and target apoptosis in tumor cells. Numerous recent studies have been conducted on TRAIL [112]. For example, Feng et al. used a targeted drug delivery system with promising results, showing that TRAIL enhanced the targeting ability of nanoparticles and acted synergistically with DOX-inhibiting tumor cells [113]. Pumilio-1 (PUM1) is an RNA-binding pumilio protein with a dramatically increased expression level in various tumor tissues. PUM1 stimulates tumor cell proliferation, motility, and colony formation in colon cancer, and is required to regulate tumor spherification [114]. TPL therapy decreased PUM1 expression in PC, activating autophagy to enhance TRAIL sensitivity in tumor cells. The data revealed potential molecular insights into TPL’s effect on drug resistance [91]. Similarly, Zhong et al. evaluated TPL’s anti-tumor efficacy against SKOV3/DDP OC cells and discovered that TPL, which induces ROS production, strongly depressed the JAK2/STAT3 signaling pathway and promoted toxic autophagy [92]. Consistent with this evidence, terpenoids such as oleanolic acid (OA) [93], AGE [94], and demethylzeylasteral (ZST93) [95] have been demonstrated to overcome drug resistance by inducing lethal autophagy and cell apoptosis. 

Resveratrol (RV) is a polyphenol compound possessing anti-tumor, anti-bacterial, anti-inflammatory, and anti-aging activities. Recently, it was discovered that RV acts as a sensitizer to chemotherapeutic agents and aids in the fight against drug resistance in human cancers [115]. RV has been implicated in the apoptosis and autophagy of tumor cells. RV activates the PI3K/AMPK signaling pathway in drug-resistant oral cancer cells, enhances the expression of autophagy-related genes, and promotes autophagic death. Notably, RV is relatively non-toxic to normal oral cells [96]. Further transcriptomic analysis revealed that RV could influence the migration of OC cells by the targeted hedgehog (Hh) pathway and epithelial–mesenchymal transition, and subsequently acts against lysophosphatidic acid (LPA, a lipid growth factor that promotes drug resistance) activity. BMI-1 plays a critical role in the hedgehog pathway. Ferraresi et al. demonstrated that treatment of RV downregulated BMI-1 expression in response to LPA treatment and restored toxic autophagy, sensitizing tumor cells to platinum-based therapy [97]. In addition to RV, structural analogs of RV have been investigated for their ability to resist drug resistance by targeting autophagy [98]. High levels of RAGE and MDR1 protein expression have been seen in GEM-resistant pancreatic ductal adenocarcinoma (PDAC) cells. RAGE activates the PI3K/AKT signaling pathway, resulting in MDR1 overexpression. The RAGE/PI3K/AKT/MDR1 axis is critical for the GEM resistance process. Piceatannol (PTE) downregulates RAGE expression, decreases the expression of p-PI3K, p-AKT, and MDR1, and enhances autophagic death, indicating a reversal of GEM resistance [98]. Furthermore, several polyphenols such as quercetin [99], hyperoside [100], scutellarin [101], and chrysin [102], which reduce off-target effects and enhance drug sensitivity, are currently under investigation.

Anthraquinones are NPs that are frequently used to treat constipation. They exert anti-inflammatory, anti-oxidative damage, and anti-tumor properties. Currently, anthraquinones have shown unique chemotherapeutic efficacy [116]. The development of nano-drug delivery technologies, in particular, has increased the bioavailability and targeting ability of anthraquinones [117,118]. Tanshinone IIA (Tan IIA), a diterpenoid quinone isolated from the botanical drug Salvia miltiorrhiza, modulates autophagy through regulating the Beclin1/LAMP1 and PI3K/Akt/mTOR signaling pathways. Tan IIA promotes autophagy and reduces ADM-induced cardiotoxicity and oxaliplatin-induced peripheral neurotoxicity [119,120]. Alternatively, Tan IIA has been utilized to address resistance to drugs such as DOX resistance. Tan IIA alone was ineffective at inhibiting the proliferation of drug-resistant GC cells. However, a Tan IIA and ADM combination showed promising toxic autophagy against drug-resistant GC cells in vitro (SNU-216, SNU-601, SNU-620, SNU-638, SNU-668, and SNU-719 cell lines, 5 µM, 24–72 h). These findings provide a theoretical basis for clinical trials combining Tan IIA and ADM to treat GC [121]. Numerous studies have demonstrated that thymoquinone (TQ) has beneficial anti-cancer properties [122]. Mifepristone is a progesterone receptor antagonist with anti-proliferative effects on endothelial cells. Mifepristone may also act as a potential chemotherapy drug sensitizer [123]. One intriguing study demonstrated that when used to treat polycystic ovarian syndrome, mifepristone may generate chronic inflammation mediated by p65 by increasing autophagy. In contrast, TQ inhibits the side effects of mifepristone by upregulating aromatase, decreasing androgen receptor expression, and reducing autophagic flux [124]. It is worth acknowledging that TQ exhibits a direct inhibitory effect on the GEM resistance of BC. Bashmail et al. showed that TQ enhanced the chemo modulatory effect of GEM by inducing autophagic cell death [103]. 

### 5.3. Natural Products with a Dual Role in Autophagy Regulation

**Figure 3:**
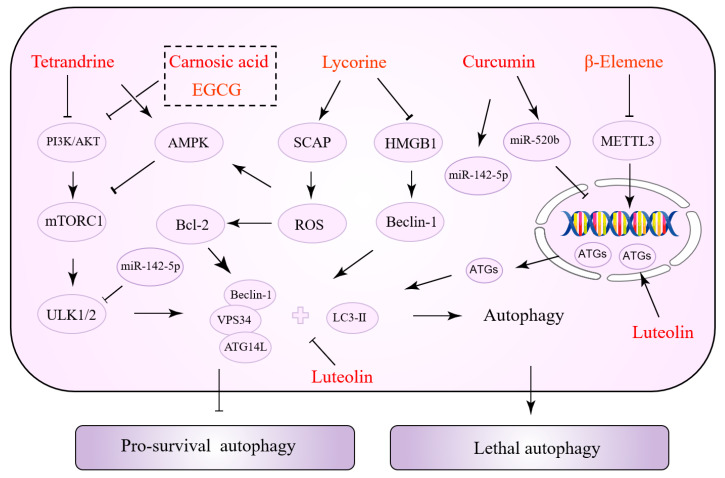
Natural products with a dual role in autophagy regulation.

Interestingly, the same NPs have various effects at different stages of tumor development. Some NPs can inhibit both autophagy to reverse drug resistance and activate autophagy to promote tumor cell apoptosis. This might be associated with the cancer type, cell line, and the duration and concentration of drug action. Table 3 summarizes the NPs exerting a dual role in autophagy regulation. Figure 3 depicts the precise process of how NPs control autophagy.

Tetrandrine (TET) has been shown to exert anti-cancer properties via targeting autophagy, according to a recent review [125]. TET induces autophagy in tumor cells by increasing the accumulation of ROS. Specifically, ROS activates ERK/MAPK and promotes the transcription of ATG7, thus promoting autophagy [126,127]. Furthermore, autophagy and apoptosis induced by TET-stimulated ROS accumulation may be controlled by caspase-3, which mediates apoptosis via interacting with p21, but not by AKT activity [128]. TET, on the other hand, can inhibit mTOR and induce autophagy by acting as a protein kinase C (PKC) inhibitor. This signaling pathway is not dependent on ROS [129]. TET promotes autophagy, which improves chemotherapeutic drug susceptibility. In NSCLC, TET and cisplatin decrease PI3K/AKT activity and Bcl-2 expression, which are important regulators of cellular autophagy inhibition [130]. In addition to the PI3K/AKT pathway, TET enhanced autophagy by downregulating the protein expression of survivin (one of the most potent anti-apoptotic suppressor genes). In turn, it reverses GEM resistance and promotes apoptosis in drug-resistant PANC-1 cells [131]. Tamoxifen (TMX) is an anti-estrogenic medication used to treat some types of breast and endometrial cancer. Wang et al. demonstrated that TET increases TMX sensitivity. In vitro experiments revealed that autophagy inhibition could be a mechanism for TET to overcome tamoxifen resistance [132]. Epidermal growth factor receptor-tyrosine kinase inhibitors (EGFR-TKIs) such as gefitinib, erlotinib, and afatinib are routinely used to treat patients with lung adenocarcinoma. However, the development of acquired drug resistance impacts the overall therapy result. By blocking lysosomes, TET improves the susceptibility of gefitinib in human lung cancer cells [133]. As previously stated, TET’s control of autophagy may be the opposite in different malignancies and drug resistance states.

**Table 3:** Natural products with a dual role in the regulation of autophagy.

Compounds	Plant Origin	Classification	Cancer Types	Models and Dosage	Mechanism	Main Effects	References
Tetrandrine	Stephania tetrandra S. Moore	Alkaloids	Non-small-cell lung cancer (NSCLC)	A549/DDP cell line (0.25 µg/mL, 12 h)	Induces autophagy and promotes cell apoptosis	Facilitates cisplatin sensitivity	[130]
			Pancreatic cancer	PANC-1 cell line (40 µg/mL, 24 h)	Induces autophagy and promotes cell apoptosis	Facilitates gemcitabine sensitivity	[131]
			Breast cancer	TAM-R and MCF-7 cell lines (1.8 µg/mL, 24 h)	Inhibits autophagy and promotes cell apoptosis	Facilitates tamoxifen sensitivity	[132]
			Lung cancer	PC14 cell line (3 µM, 72 h)	Inhibits autophagy	Facilitates gefitinib sensitivity	[133]
Lycorine	Amaryllidaceae	Alkaloids	Multiple myeloma	ANBL6, ARP-1, ARH-77, H929, and MM.1S cell lines (10 µM, 24 h)	Inhibits autophagy and promotes cell apoptosis	Facilitates bortezomib sensitivity	[134]
			Hepatocellular carcinoma (HCC)	In vitro: PLC/PRF/5 and MHCC-97H cell lines (1, 2, 4 µmol/L) In vivo: mice (oral gavage, 10 mg/kg/day)	Induces autophagy and promotes cell apoptosis	Facilitates sorafenib sensitivity	[135]
Carnosic acid	Rosmarinus officinalis/Salvia officinalis	Terpenoids	Glioma	U251 and LN229 cell lines (10 µM, 24 h)	Induces autophagy and promotes cell apoptosis	Facilitates temozolomide sensitivity	[136]
			Hepatocellular carcinoma	Huh7 and HCO2 cell lines (10 µM, 48 h)	Induces autophagy and promotes cell apoptosis	Facilitates sorafenib sensitivity	[137]
			Breast cancer	SKBR-3, BT474, MCF7, and MDA-MB-231 cell lines (27.5, 37.5 µM, 48 h)	Inhibits autophagy and promotes cell apoptosis	Facilitates trastuzumab sensitivity	[138]
β-Elemene	Curcuma longa L.	Terpenoids	Colorectal cancer	In vitro: HCT116p53 +/+ and HCT116p53 –/– cell line (40 µg/mL, 24 h) In vivo: mice (intraperitoneally injected, 100 mg/kg/day)	Induces autophagy	Facilitates 5-FU sensitivity	[139]
			NSCLC	In vitro: PC9GR and HCC827GR cell lines (120 µg/mL, 24 h) In vivo: mice (intraperitoneally injected, 100 mg/kg/day)	Inhibits autophagy and promotes cell apoptosis	Facilitates gefitinib sensitivity	[140]
Curcumin	Rosmarinus officinalis/Salvia officinalis	Polyphenols	NSCLC	A549, H460, H1299, and H1066 cell lines (10–30 µM, 48 h)	Inhibits autophagy and promotes cell apoptosis	Facilitates grizotinib sensitivity	[141]
			NSCLC	In vitro: H157, H1299, and PC9 cell lines (5 µM, 48 h) In vivo: mice (oral gavage, 1 g/kg/day)	Induces autophagy and promotes cell apoptosis	Facilitates gefitinib sensitivity	[142]
Luteolin		Polyphenols	HCC	Huh7 and Hep3B cell lines (20 µM, 18 h)	Induces autophagy and promotes cell apoptosis	Facilitates TNF-related apoptosis-inducing ligand (TRAIL) sensitivity	[143]
			Ovarian cancer		Inhibits autophagy and promotes cell apoptosis	Facilitates cisplatin sensitivity	[138]
Epigallocatechin gallate	Camellia sinensis	Polyphenols	Oral cancer	SKVCR cell line (10–75 µM, 24 h)	Induces autophagy and promotes cell apoptosis	Facilitates cisplatin sensitivity	[144]
			NSCLC	In vitro: A549 cell line (34 µM, 48 h) In vivo: mice (oral gavage, 200 mg/kg/day)	Inhibits autophagy and promotes cell apoptosis	Facilitates gefitinib sensitivity	[145]

Lycorine is an active alkaloid isolated from a plant in the amaryllidaceae family that has received increasing attention from researchers because of its anti-cancer properties [146]. Recently, Hu et al. devised and constructed a novel nanocomposite comprising lycorine and folic-acid-modified mesoporous silica-coated gold nanostars [147]. The mass release of lycorine once the nanocomposite is internalized into cancer cells causes mitochondrial dysfunction and ERS in tumor cells. It causes an increase in ROS, which leads to apoptosis, and notably, a higher inhibitory effect and selectivity against drug resistance. Tongue-cancer-resistant protein 1 (TCRP1) may be a candidate oncoprotein that promotes chemotherapy resistance in tumors and is highly linked to TMX, cisplatin, and radiation resistance. Previous studies have revealed that TCRP1 is a target gene for c-Myc [148], a protein that regulates the phosphoinositide-dependent kinase 1 (PDK1)/serum- and glucocorticoid-inducible kinase 1 (SGK1) [149] and PI3K/AKT/NF-κB [150] signaling pathways, promoting TCRP1 transcription to render tumor cells resistant to chemotherapeutic agents. Lycorine has recently been shown to be a possible TCRP1 inhibitor [151]. The TCRP1 protein degradation pathway in hepatocellular carcinoma (HCC) cells results in the suppression of the AKT/mTOR signaling pathway and an increase in autophagic flux. Lycorine-induced c-Myc inhibition increases the TCRP1 protein degradation pathway [152]. Proteasome inhibitors such as bortezomib, carfilzomib, and isazomib are effective in the treatment of multiple myeloma (MM). However, some patients have various degrees of side effects and drug resistance. HMGB1 is a critical signaling molecule in autophagy. Bortezomib-resistant myeloma cells are more responsive to bortezomib because lycorine blocks HMGB1-mediated autophagy, according to Roy et al. [134]. The sterol regulatory element-binding protein (SREBP) cleavage-activating protein (SCAP) is a sterol-sensitive protein that regulates triglyceride and cholesterol levels. In a recent study, SCAP was shown to be substantially expressed in HCC tissues and associated with sorafenib resistance. Lycorine, a specific SCAP inhibitor, triggered autophagy by elevating AMPK activity, increasing the HCC cells’ sensitivity to sorafenib [135].

Carnosic acid (CA) is a polyphenolic diterpene that has recently been found to be helpful against tumor drug resistance. CA overcomes TMZ resistance in glioma cancer cells by the induction of autophagy; CA directly inhibits the PI3K/AKT signaling pathway and induces autophagy by interacting with p62 [136]. In addition, CA has a direct synergistic effect on the vitamin D2 analog doxercalciferol (D2), enhancing sorafenib-mediated tumor cell death. Increased cytotoxic autophagy is associated with an increase in this efficacy. After the treatment of HCC cells with a CA and Vitamin D2 analog combination, an increase in cytoplasmic vacuolization was seen, along with increased protein levels of Beclin1, ATG3, and LC3 [137]. Trastuzumab (Tz), a monoclonal antibody directed against the human epidermal growth factor receptor 2 (HER-2), dramatically improves the prognosis of BC patients who are HER-2-positive. CA partially restores the BC cells’ susceptibility to Tz by inhibiting autophagy [138]. β-Elemene is another terpene that enhances the sensitivity of cancer cells to particular medicines. Numerous evidence supports the notion that, in terms of autophagy, β-elemene increases p53-induced toxic autophagy and cyclin D3-dependent cycle arrest to reverse 5-FU resistance [139]. It has been shown that β-elemene inhibits the inhibition of autophagic flux in gefitinib-resistant cells while also decreasing the expression of methyltransferase-like 3 (METTL3). METTL3 promotes autophagy by increasing the expression of ATG5 and ATG7 [140].

Curcumin (CUR) is critical for overcoming drug resistance in tumors via various mechanisms including decreased drug uptake, drug efflux, PCD, epigenetics, and DNA damage responses [14]. CUR may also be a potential autophagy modulator [153]. As small non-coding RNAs with regulatory functions, some miRNAs govern cellular autophagy and are involved in tumor cell proliferation, migration, invasion, and drug resistance. miR-142-5p is the highlight of this series of studies, demonstrating the suppressive efficacy of chemotherapy drug resistance [154]. CUR inhibits miR-142-5p and its target ULK1 on autophagy, reducing crizotinib resistance in NSCLC cells [141]. It is concerned with CUR’s synergistic effect on gefitinib. By disrupting the interaction of Sp1 and HADC1, CUR and gefitinib induce autophagic cell death by downregulating EGFR activity and inhibiting the ERK/MEK and AKT/S6K pathways. CUR’s sensitizing impact on gefitinib was abolished by autophagy inhibitors or the inhibition of Beclin-1 or ATG7. CUR and gefitinib effectively suppress tumor growth in xenograft experiments [142]. Luteolin exhibits multiple biological activities and attacks tumor cells by diverse mechanisms. First, luteolin inhibits HIF-1 activity and modulates autophagy, which aids in the fight against hypoxic tumors [155]. In addition, luteolin enhances TRAIL-induced apoptosis by increasing autophagic flux. The toxic autophagy of tumor cells was induced by the upregulation of DR5 expression. The c-Jun N-terminal kinase (JNK) inhibitors inhibit DR5, nullifying the toxic effects of luteolin [143]. In contrast, luteolin inhibits autophagy in OC cells and enhanced cisplatin sensitivity by suppressing PARP1 expression [156]. In conclusion, through modulating autophagy, lignocaine exerts a durable synergistic effect against drug resistance. Epigallocatechin gallate (EGCG) is a key component of catechins with a wide range of biological properties. Previous studies have shown that EGCG has a considerably detrimental effect on cisplatin-resistant oral cancer CAR cells via a mechanism that may be related to autophagy and apoptosis mediated by the AKT/STAT3 signaling pathway [144]. Later, Jiao et al. found a link between EGCG and autophagy in tumor drug resistance. EGCG suppresses tumor cell autophagy by targeting the ERK signaling pathway, eliminating gefitinib resistance in NSCLC cells [145].

The NPs andrographolide [59], α-hederin [62], jolkinolide B [63], PC3-15 [64], pristimerin [65], icariin [67], apigenin [69], tea polyphenol [70], genistein [71], phloretin [72], FMNT [73], rutin [74], berberine [86,87], matrine [88], ursolic acid [104], betulinic acid [90], triptolide [92], oleanolic acid [93], AGE [94], demethylzeylasteral [95], resveratrol [96], piceatannol [98], quercetin [99], hyperoside [100], scutellarin [101], chrysin [102], thymoquinone [103], tetrandrine [126,127], lycorine [134], carnosic acid [137], β-elemene [140], curcumin [141], luteolin [155], and epigallocatechin gallate [145] are associated with autophagy in tumor cells. These NPs can control autophagy in tumor cells such as those of the liver, pancreatic, gastric, breast, myeloma, lung, glioma, colorectal, ovarian, cervical, and oral malignancies, and thus combat drug resistance. In particular, some NPs combat tumor drug resistance by reducing protective autophagic flux. Conversely, NPs that induce autophagic cell death may increase anticancer drug sensitivity by directly promoting tumor cell death or commencing a regulatory mechanism. In conclusion, the investigation of NPs that regulate autophagy to overcome drug resistance in tumors may provide new therapeutic approaches for cancer treatment.

## 6. Limitations

However, issues in the research process continue to be a “stumbling obstacle” for NPs sought for application in the clinic. NPs including alkaloids, terpenoids, polyphenols, and anthraquinones regulate autophagy by influencing numerous signaling pathways including PI3K/AKT/mTOR, ERK, JNK, and AMPK [157,158,159,160]. On one hand, most studies on the mechanisms of NPs in the regulation of autophagy are still at an early stage. The mode of interaction between NPs and autophagy-related targets remains to be elucidated. On the other hand, the signals above-mentioned do not exist in isolation, but overlap and constitute a regulatory network affecting tumor drug resistance. However, most current research focuses on a few targets or single pathways, although less research has been conducted on the multi-target, multi-pathway, and integrated control mechanisms of NPs.

Furthermore, throughout the therapeutic process, certain NPs may either stimulate or inhibit autophagy. This is a distinct benefit of NPs, because fighting drug resistance requires bidirectional modulation of autophagy. However, the studies summarized in this paper are unidirectional experiments in which NPs affect autophagy, and the kind of cancer, cell line, dosage, and administration time vary. As a result, the “bidirectional control” of NPs in tumor cells remains controversial.

## 7. Conclusions

Cancer is a global medical challenge that presents grave risks to human health. Both the incidence and death rates of cancer are on the rise. There were approximately 19.3 million new cancer diagnoses and 9.9 million cancer-related deaths worldwide in 2020. Even though chemotherapy and targeted treatments provide some patients with a glimmer of hope, drug resistance has become the most challenging obstacle in cancer treatment. Numerous studies have demonstrated that combining NPs such as alkaloids, terpenoids, polyphenols, and anthraquinones with anti-cancer drugs can result in tumor cells becoming sensitized to anti-tumor drugs, increasing their effectiveness. To be more precise, both inducers and inhibitors of autophagy can be utilized to overcome drug resistance. Anti-tumor drugs can benefit from NPs that block protective autophagy or induce autophagic cell death. Compared with CQ or HCQ, several NPs have also been shown to dramatically minimize the hazardous side effects of chemotherapy medications.

There are also a few obstacles worth mentioning. Autophagic flux and indicators of autophagy have not been consistently measured or identified across investigations. The autophagy cascade, in particular, is an ever-changing mechanism. It is also problematic that few studies have only explored the influence of NPs on autophagy at a specific moment in time, which might cause bias. The experimental outcomes of numerous studies differ, which might cause problems when comparing and assessing therapeutic effectiveness. Finally, the signaling molecules implicated in botanical-drug-regulated autophagy against drug resistance interact and intersect. Future research should focus on numerous signaling pathways rather than just one.

In summary, resistance to chemotherapeutic and synthetic drugs is a complex process. Despite the significant synergistic benefits of NPs, many preclinical and clinical studies are required to establish their value.

## Data Availability

Not applicable.
